# Neurostructural Differences Associated With Prodromal Mania Symptoms in Children

**DOI:** 10.1002/brb3.70894

**Published:** 2025-09-21

**Authors:** Camille Archer, Amy Milewski, Hee Jung Jeong, Gabrielle E. Reimann, E. Leighton Durham, Antonia N. Kaczkurkin

**Affiliations:** ^1^ Department of Psychology Vanderbilt University Nashville Tennessee USA; ^2^ Department of Psychology Sogang University Seoul South Korea

**Keywords:** brain structure, children, gray matter volume, mania, pathophysiology

## Abstract

**Introduction:**

Prodromal symptoms of mania in children are predictive of the later development of bipolar disorder; yet, the neurostructural correlates of these early symptoms remain poorly understood. This study aimed to investigate the association between prodromal mania symptoms and brain structure in a large cohort of children.

**Methods:**

We analyzed data from 10,662 nine‐ to 10‐year‐old children from the Adolescent Brain Cognitive Development (ABCD) Study, employing structural equation modeling to examine the concurrent and longitudinal associations between prodromal mania symptoms and cortical and subcortical gray matter volume.

**Results:**

After adjusting for multiple comparisons and controlling for age, sex, scanner model, socioeconomic status, and medication use, we found that baseline mania symptoms were associated with reduced gray matter volume across both cortical and subcortical areas, suggesting a global effect. These findings were further supported by the loss of these effects when total intracranial volume was included as an additional covariate, suggesting that smaller overall brain size, rather than specific regional effects, is related to prodromal mania symptoms. Lastly, longitudinal analyses revealed that brain volume at baseline did not predict prodromal mania symptoms at the second‐year follow‐up.

**Conclusion:**

Our results support the structural differences observed in adults with bipolar disorder in prior work and refine our understanding of the neurostructural correlates of prodromal mania symptoms in children. These findings could enhance early identification and intervention efforts for youth at risk of developing bipolar disorder.

## Graphical abstract

The current study examined the relationship between prodromal mania symptoms and brain structure in a large sample of children. We found that baseline mania symptoms were associated with smaller brain volumes in all cortical and subcortical regions, suggesting a global effect that is also supported by the loss of these effects when total intracranial volume was included as an additional covariate. These results help to refine our understanding of the neurostructural correlates of mania symptoms during the prodromal stage, which could be leveraged for improved identification and intervention.

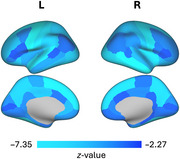

## 1 Introduction

Bipolar disorder is a severe mental health condition marked by alternating periods of mania and depression and often leads to an increased risk of suicide, as well as cognitive and functional impairments that worsen over time (Lan et al. 2014; Maletic and Raison 2014; Muneer 2016). Although the mean age of onset for the first manic, hypomanic, or depressive episode is around 18 years for bipolar I disorder and in the mid‐20s for bipolar II disorder (American Psychiatric Association 2022), bipolar spectrum disorders (including bipolar I, bipolar II, cyclothymic disorder, and other specified or unspecified bipolar disorders) have also been observed in children and adolescents (Goldstein et al. 2017). Diagnosing bipolar spectrum disorders in youth is challenging, often resulting in children and adolescents receiving a diagnosis of other specified bipolar and related disorder (Goldstein et al. 2017). Nevertheless, a significant portion of children and adolescents initially diagnosed with other specified bipolar and related disorder eventually develop bipolar I or II, suggesting that prodromal mania symptoms may serve as early indicators of the disorder (Axelson et al. 2011; Conroy et al. 2018). Prodromal mania symptoms, such as excitation, mood swings, irritability, anxiety, hyperactivity, and sleep disturbances, can manifest months or years before the first full manic or hypomanic episode, which underscores the importance of early detection and intervention (Correll et al. 2014; De Pablo et al. 2020; Skjelstad et al. 2010). Despite growing awareness of the importance of detecting prodromal mania symptoms in youth, there is a lack of research on the neurostructural correlates of these early symptoms.

Neurostructural differences are evident in individuals with bipolar disorder throughout the lifespan. In adults with bipolar disorder, research consistently shows cortical alterations in frontal, temporal, and parietal brain regions (Abé et al. 2016; Hibar et al. 2018). Specifically, adults with bipolar disorder present with smaller gray matter volume in the dorsomedial and ventromedial prefrontal cortex, anterior cingulate cortex, bilateral insula, and superior temporal gyrus (Wang et al. 2019; Wise et al. 2017). Additionally, smaller gray matter volumes in the amygdala and hippocampus are related to bipolar disorder in adult populations (Angelescu et al. 2021; Hibar et al. 2016). In contrast, adults with bipolar disorder show greater gray matter volume in areas such as the inferior temporal gyrus and bilateral middle frontal gyrus, as well as cerebellar regions (Wise et al. 2017).

When examining structural differences in youth with bipolar disorder, high‐risk children and adolescents show smaller cortical gray matter volume in frontal regions, including the inferior frontal gyrus, lateral orbitofrontal cortex, frontal pole, and rostral middle frontal gyrus (Lim et al. 2013; Roberts et al. 2022). Bilateral amygdala and limbic region volumes are also smaller in adolescents with bipolar disorder, with longitudinal studies showing that amygdala volume reduction is specific to youth compared to adults (Förster et al. 2023; Long et al. 2023). In contrast, some work has found that youth with bipolar disorder show *larger* volumes in the right medial orbitofrontal cortex and left superior frontal gyrus (Long et al. 2023; Roberts et al. 2022). Additionally, children with bipolar disorder have been shown to have overall smaller total cerebral brain volume in some studies (Frazier, Ahn, et al. 2005).

Taken together, the existing literature highlights both distinct and shared neurostructural differences in adults and children with bipolar disorder. However, much of the research on structural differences in youth has focused on pediatric or adolescent samples with a diagnosis of a bipolar spectrum disorder (Dickstein et al. 2005; Frazier, Breeze, et al. 2005; Gogtay et al. 2007; Kaur et al. 2005; Toma et al. 2019). Given that subthreshold manic symptoms are often present for a lengthy period of time before the first full manic or hypomanic episode (Correll et al. 2014; Van Meter et al. 2021), studies are needed that examine the neurostructural correlates of prodromal mania symptoms in children *prior* to receiving a bipolar disorder diagnosis. Furthermore, many of the studies on structural differences in children with bipolar disorder have relied on relatively small samples sizes with varying age ranges. Research involving a large cohort of children with a narrow age range would be useful to determine whether neural correlates of prodromal mania symptoms can be detected before the onset of the first manic or hypomanic episode.

The current study aimed to overcome these previous limitations by exploring the relationship between a dimensional measure of prodromal mania symptoms and regional cortical and subcortical brain volumes in children. We examined this association in a large, community‐based sample of 9‐ to 10‐year‐olds (*N* = 10,662) at baseline and again over a 2‐year period. We hypothesized that higher levels of prodromal mania symptoms would be associated with smaller brain volume in areas previously implicated in bipolar disorder, including frontal regions and the amygdala. Additionally, we predicted that smaller brain volume in these regions at baseline would predict higher levels of prodromal mania symptoms at the second‐year follow‐up.

## 2 Methods and Materials

### 2.1 Participants

This study drew on baseline and second‐year follow‐up data from the Adolescent Brain Cognitive Development (ABCD) Study, release 5.1 (Volkow et al. 2018). Recruitment for the ABCD Study took place in the United States across 21 sites, with the baseline sample size consisting of data from 11,868 children aged 9–10 years old (Casey et al. 2018; Volkow et al. 2018). The current analyses utilized data from 10,662 participants after excluding those with missing data or who failed to pass quality assurance measures. Table 1 provides further details on the final sample's demographic information. Vanderbilt University's Institutional Review Board approved the use of this publicly available, de‐identified dataset. Caregivers in the ABCD Study provided informed consent and minors provided informed assent.

**TABLE 1:** Demographics of the sample (*N* = 10,662).

		Mean	*SD*
Age (years)		9.92	0.63
		*N*	*%*
Sex			
	Female	5168	48.47
	Male	5494	51.53
Race/ethnicity			
	White	5594	52.47
	Hispanic	2186	20.50
	African American	1542	14.46
	Other	1340	12.57
Household annual income			
	< $5000	346	3.25
	$5000–$11,999	371	3.48
	$12,000–$15,999	253	2.37
	$16,000–$24,999	471	4.42
	$25,000–$34,999	585	5.49
	$35,000–$49,999	830	7.79
	$50,000–$74,999	1357	12.73
	$75,000–$99,999	1430	13.41
	$100,000–$199,999	2995	28.09
	≥$200,000	1133	10.62
	Missing	891	8.35
Parental education			
	No degree	532	4.99
	Highschool degree/GED	1291	12.11
	Some college	1742	16.34
	Associate's degree	1385	12.99
	Bachelor's degree	3010	28.23
	Master's degree	2054	19.26
	Professional/doctoral degree	648	6.08

*Note*: The “Other” race/ethnicity category includes those who were identified by their parent as American Indian/Native American, Alaska Native, Native Hawaiian, Guamanian, Samoan, Other Pacific Islander, Asian Indian, Chinese, Filipino, Japanese, Korean, Vietnamese, Other Asian, or Other Race.

Abbreviation: *SD*, standard deviation.

### 2.2 Measures

#### 2.2.1 Prodromal Mania Measure

The Parent General Behavior Inventory, 10‐Item Mania scale (PGBI‐10 M) was developed from the original 73‐item PGBI and includes the 10 most predictive items for assessing prodromal bipolar disorder symptoms, including manic and biphasic (mixed state including aspects of both mania and depression) symptoms (Freeman et al. 2012; Youngstrom et al. 2008). This caregiver‐rated measure has been shown to distinguish prodromal bipolar disorder symptoms from comorbid conditions such as unipolar depression and attention‐deficit/hyperactivity disorder (ADHD) (Barch et al. 2018; Youngstrom et al. 2008). The PGBI‐10 M shows excellent internal consistency (*α* = 0.92) and high correlations with earlier versions in prior work (Youngstrom et al. 2008).

#### 2.2.2 Image Acquisition, Quality Assurance, and Processing

The ABCD Study collected MRI data on multiple models of 3 tesla (3T) scanners: General Electric Discovery MR750, Siemens Prisma, Siemens Prisma Fit, Philips Achieva dStream, and Philips Ingenia (Casey et al. 2018). Three‐dimensional T1‐ and T2‐weighted images of brain structure were collected, with whole brain T1‐weighted images obtained using the following parameters: TR (repetition time) 2400–2500 ms; TE (echo time) 2–2.9 ms; FOV (field of view) 256 × 240 to 256; FOV phase of 93.75%–100%; matrix 256 × 256; 176–225 slices; TI (inversion delay) 1060 ms; flip angle of 8°; and voxel resolution of 1 × 1 × 1 mm; total acquisition time was 7 min and 12 s for Siemens Prisma, 6 min and 9 s for GE 750, and 5 min and 38 s for Philips.

The imaging data were processed and analyzed by the ABCD Data Analysis and Informatics Center (DAIC) using the Multi‐Modal Processing Stream (MMPS), a software package created at the Center for Multimodal Imaging and Genetics (CMIG) at the University of California, San Diego (UCSD). Data underwent correction for gradient nonlinearity distortions, intensity scaling and homogeneity correction, registration to an averaged reference brain in standard space, and manual quality control. Using automated, atlas‐based, segmentation processes in FreeSurfer v.5.3, data underwent cortical surface reconstruction and subcortical segmentation. To quantify morphometric measures, DAIC used the average cortical thickness and volume in 68 cortical parcels of the Desikan–Killiany atlas (Desikan et al. 2006) and the average volume in 19 subcortical regions from the FreeSurfer subcortical atlas (Fischl et al. 2002). Trained technicians completed manual quality control on the images for motion, intensity homogeneity, white matter underestimation, pial overestimation, and magnetic susceptibility artifact. Additional details regarding the ABCD Study imaging procedures have been previously documented (Casey et al. 2018; Hagler et al. 2019).

### 2.3 Data Analysis

Utilizing structural equation modeling in Mplus version 8.8., brain volume was related to a dimensional measure of prodromal mania symptoms while controlling for age, sex, scanner model, socioeconomic status (SES; defined as parent's highest level of education), and medication use. For longitudinal analyses, baseline prodromal mania symptoms were added as a covariate. All analyses clustered based on family to account for twins and siblings and stratified based on site. Poststratification weights provided by the ABCD Study were also applied to account for discrepancies between the sample and the US population on key demographics, such as sex and race/ethnicity. As we have done in our previous work, nonparticipation weights were used to make the included and excluded samples more similar, given that participants who were included in the MRI subsample differ significantly on important demographics from those who were excluded for MRI data quality issues (Durham et al. 2021). The false discovery rate (FDR) was used to adjust *p*‐values to account for multiple comparisons.

### 2.4 Sensitivity Analyses

To further investigate the robustness of the primary findings, total intracranial volume was added as an additional covariate. This allows us to account for potential associations that may exist between brain volume and overall variations in cranium size. By including total intracranial volume in our analyses, we aimed to determine whether any regional volume effects exist, above and beyond global differences in brain size.

### 2.5 Data and Code Availability

The National Institute of Mental Health Data Archive (https://nda.nih.gov/abcd) provides access to the ABCD Study data. To access the current analyses’ scripts with a detailed description, visit https://github.com/VU‐BRAINS‐lab/Archer_Mania_Volume.

## 3 Results

### 3.1 Prodromal Mania Is Associated With Differences in Brain Structure at Baseline

**FIGURE 1:**
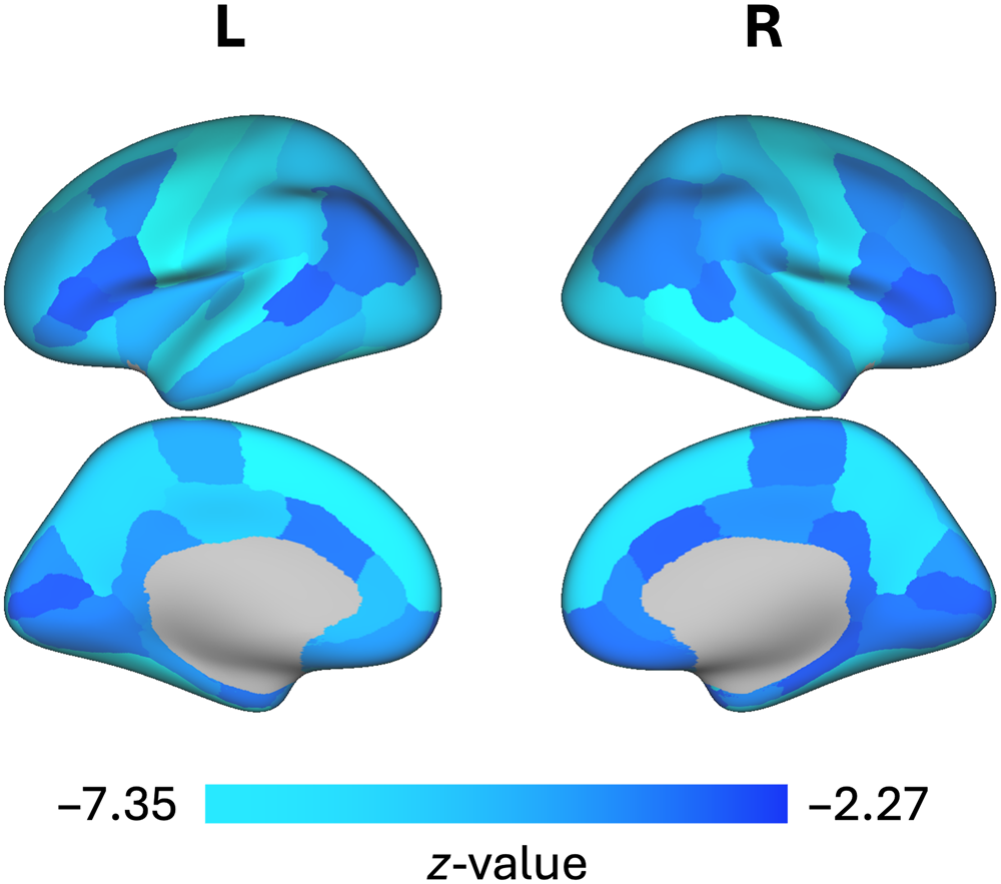
Regions with significant associations between cortical volume and prodromal mania. Higher prodromal mania scores were associated with significantly smaller cortical gray matter volumes across the brain. No regions remained significant after the addition of total intracranial volume as an additional covariate during sensitivity analyses, suggesting a global effect.

Following FDR correction for multiple comparisons and controlling for age, sex, differences in scanner model, SES, and medication use, greater prodromal mania symptoms were associated with smaller cortical and subcortical gray matter volumes in all brain regions examined (*p*_fdr_‐values ≤0.05) (Table 2). Of these regions, several demonstrated a strong association with prodromal mania symptoms, including the left fusiform gyrus, bilateral precentral gyrus, right middle temporal gyrus, and bilateral thalamus (see Figure 1).

**TABLE 2:** Associations between prodromal mania symptoms and gray matter volume in 68 cortical regions and 19 subcortical regions.

	*β*	*z*	*p*_fdr_
Left banks of superior temporal sulcus	−0.035	−2.93	0.003
Left caudal anterior cingulate	−0.042	−3.75	< 0.001
Left caudal middle frontal	−0.052	−4.44	< 0.001
Left cuneus	−0.048	−4.40	< 0.001
Left entorhinal	−0.043	−3.74	< 0.001
Left fusiform	−0.086	−7.13	< 0.001
Left inferior parietal	−0.042	−3.67	< 0.001
Left inferior temporal	−0.069	−5.83	< 0.001
Left isthmus cingulate	−0.053	−4.50	< 0.001
Left lateral occipital	−0.076	−6.23	< 0.001
Left lateral orbitofrontal	−0.074	−6.10	< 0.001
Left lingual	−0.049	−4.07	< 0.001
Left medial orbitofrontal	−0.060	−4.78	< 0.001
Left middle temporal	−0.061	−5.03	< 0.001
Left parahippocampal	−0.053	−4.72	< 0.001
Left paracentral	−0.057	−5.03	< 0.001
Left pars opercularis	−0.037	−3.10	0.002
Left pars orbitalis	−0.051	−4.22	< 0.001
Left pars triangularis	−0.027	−2.27	0.024
Left pericalcarine	−0.032	−2.71	0.007
Left postcentral	−0.080	−6.70	< 0.001
Left posterior cingulate	−0.069	−5.99	< 0.001
Left precentral	−0.086	−7.35	< 0.001
Left precuneus	−0.073	−6.18	< 0.001
Left rostral anterior cingulate	−0.062	−5.56	< 0.001
Left rostral middle frontal	−0.073	−5.97	< 0.001
Left superior frontal	−0.079	−6.87	< 0.001
Left superior parietal	−0.069	−6.05	< 0.001
Left superior temporal	−0.078	−6.79	< 0.001
Left supramarginal	−0.075	−6.12	< 0.001
Left frontal pole	−0.037	−3.13	0.002
Left temporal pole	−0.055	−4.75	< 0.001
Left transverse temporal	−0.065	−5.71	< 0.001
Left insula	−0.065	−5.20	< 0.001
Right banks of superior temporal sulcus	−0.045	−3.98	< 0.001
Right caudal anterior cingulate	−0.041	−3.35	0.001
Right caudal middle frontal	−0.050	−4.58	< 0.001
Right cuneus	−0.055	−4.81	< 0.001
Right entorhinal	−0.056	−4.69	< 0.001
Right fusiform	−0.078	−6.65	< 0.001
Right inferior parietal	−0.057	−4.47	< 0.001
Right inferior temporal	−0.087	−6.99	< 0.001
Right isthmus cingulate	−0.043	−3.64	< 0.001
Right lateral occipital	−0.081	−6.68	< 0.001
Right lateral orbitofrontal	−0.071	−5.80	< 0.001
Right lingual	−0.049	−4.05	< 0.001
Right medial orbitofrontal	−0.047	−3.98	< 0.001
Right middle temporal	−0.089	−7.35	< 0.001
Right parahippocampal	−0.038	−3.20	0.001
Right paracentral	−0.047	−4.16	< 0.001
Right pars opercularis	−0.050	−4.35	< 0.001
Right pars orbitalis	−0.059	−5.06	< 0.001
Right pars triangularis	−0.028	−2.49	0.013
Right pericalcarine	−0.041	−3.38	0.001
Right postcentral	−0.073	−6.13	< 0.001
Right posterior cingulate	−0.060	−4.72	< 0.001
Right precentral	−0.084	−6.92	< 0.001
Right precuneus	−0.077	−6.53	< 0.001
Right rostral anterior cingulate	−0.053	−4.47	< 0.001
Right rostral middle frontal	−0.056	−4.73	< 0.001
Right superior frontal	−0.076	−6.64	< 0.001
Right superior parietal	−0.068	−5.89	< 0.001
Right superior temporal	−0.066	−5.82	< 0.001
Right supramarginal	−0.057	−4.97	< 0.001
Right frontal pole	−0.053	−4.46	< 0.001
Right temporal pole	−0.038	−3.38	0.001
Right transverse temporal	−0.062	−5.77	< 0.001
Right insula	−0.082	−6.75	< 0.001
Left cerebellum cortex	−0.088	−6.82	< 0.001
Left thalamus proper	−0.099	−7.79	< 0.001
Left caudate	−0.070	−6.11	< 0.001
Left putamen	−0.061	−4.70	< 0.001
Left pallidum	−0.058	−4.56	< 0.001
Brain stem	−0.079	−5.81	< 0.001
Left hippocampus	−0.070	−5.57	< 0.001
Left amygdala	−0.064	−5.20	< 0.001
Left accumbens area	−0.055	−4.56	< 0.001
Left ventral diencephalon	−0.087	−6.70	< 0.001
Right cerebellum cortex	−0.087	−6.62	< 0.001
Right thalamus proper	−0.092	−7.34	< 0.001
Right caudate	−0.060	−5.15	< 0.001
Right putamen	−0.069	−5.21	< 0.001
Right pallidum	−0.058	−4.59	< 0.001
Right hippocampus	−0.072	−5.80	< 0.001
Right amygdala	−0.054	−4.39	< 0.001
Right accumbens area	−0.058	−4.85	< 0.001
Right ventral diencephalon	−0.079	−6.22	< 0.001

### 3.2 Total Intracranial Volume Sensitivity Analyses Reveal a Global Effect

Sensitivity analyses were conducted with total intracranial volume included as an additional covariate, alongside age, sex, scanner model, SES, and medication use. Although the main analyses showed an association between higher prodromal mania scores and smaller cortical and subcortical volumes at baseline, this relationship was no longer significant after controlling for intracranial volume. The inclusion of total intracranial volume resulted in the disappearance of significant effects in all brain regions tested (*p*_fdr_‐values ≥0.07) (Table 3).

**TABLE 3:** Associations between prodromal mania symptoms and gray matter volume in 68 cortical regions and 19 subcortical regions with total intracranial volume as an additional covariate.

	*β*	*z*	*p*	*p*_fdr_
Left banks of superior temporal sulcus	−0.017	−1.66	0.096	0.363
Left caudal anterior cingulate	0.003	0.33	0.744	0.830
Left caudal middle frontal	−0.027	−2.68	0.007	0.203
Left cuneus	−0.014	−1.31	0.190	0.384
Left entorhinal	−0.015	−1.49	0.136	0.377
Left fusiform	−0.015	−1.33	0.185	0.383
Left inferior parietal	−0.012	−1.16	0.247	0.467
Left inferior temporal	−0.014	−1.23	0.218	0.431
Left isthmus cingulate	−0.025	−2.44	0.015	0.203
Left lateral occipital	−0.009	−0.88	0.379	0.525
Left lateral orbitofrontal	−0.010	−0.88	0.379	0.525
Left lingual	−0.025	−2.41	0.016	0.203
Left medial orbitofrontal	−0.010	−0.87	0.386	0.525
Left middle temporal	−0.014	−1.34	0.181	0.383
Left parahippocampal	−0.002	−0.21	0.836	0.909
Left paracentral	−0.014	−1.41	0.160	0.377
Left pars opercularis	0.009	0.83	0.408	0.539
Left pars orbitalis	0.012	1.10	0.270	0.475
Left pars triangularis	0.005	0.48	0.633	0.741
Left pericalcarine	−0.020	−1.88	0.060	0.274
Left postcentral	−0.015	−1.45	0.148	0.377
Left posterior cingulate	−0.016	−1.59	0.113	0.377
Left precentral	−0.019	−1.76	0.079	0.312
Left precuneus	−0.010	−0.91	0.362	0.525
Left rostral anterior cingulate	−0.011	−1.13	0.257	0.475
Left rostral middle frontal	−0.010	−0.89	0.376	0.525
Left superior frontal	−0.028	−2.68	0.007	0.203
Left superior parietal	−0.001	−0.06	0.953	0.975
Left superior temporal	−0.014	−1.39	0.165	0.377
Left supramarginal	−0.009	−0.81	0.415	0.539
Left frontal pole	0.001	0.08	0.934	0.967
Left temporal pole	−0.007	−0.71	0.478	0.597
Left transverse temporal	−0.018	−1.86	0.063	0.274
Left insula	−0.013	−1.20	0.229	0.443
Right banks of superior temporal sulcus	−0.011	−1.00	0.316	0.525
Right caudal anterior cingulate	−0.011	−1.10	0.273	0.475
Right caudal middle frontal	−0.005	−0.47	0.640	0.741
Right cuneus	−0.014	−1.41	0.158	0.377
Right entorhinal	−0.017	−1.61	0.107	0.377
Right fusiform	−0.023	−2.25	0.025	0.203
Right inferior parietal	−0.011	−1.01	0.314	0.525
Right inferior temporal	−0.016	−1.51	0.131	0.377
Right isthmus cingulate	0.000	0.01	0.990	0.990
Right lateral occipital	−0.016	−1.46	0.145	0.377
Right lateral orbitofrontal	−0.012	−1.11	0.266	0.475
Right lingual	−0.022	−2.20	0.028	0.203
Right medial orbitofrontal	−0.023	−2.29	0.022	0.203
Right middle temporal	−0.008	−0.71	0.475	0.597
Right parahippocampal	−0.007	−0.70	0.487	0.597
Right paracentral	−0.007	−0.70	0.486	0.597
Right pars opercularis	−0.004	−0.41	0.684	0.773
Right pars orbitalis	−0.005	−0.49	0.623	0.741
Right pars triangularis	−0.002	−0.19	0.852	0.915
Right pericalcarine	−0.014	−1.40	0.163	0.377
Right postcentral	0.000	−0.02	0.981	0.990
Right posterior cingulate	−0.009	−0.82	0.414	0.539
Right precentral	−0.015	−1.38	0.169	0.377
Right precuneus	−0.010	−0.95	0.341	0.525
Right rostral anterior cingulate	−0.015	−1.42	0.156	0.377
Right rostral middle frontal	−0.020	−1.89	0.059	0.274
Right superior frontal	−0.025	−2.26	0.024	0.203
Right superior parietal	−0.009	−0.90	0.369	0.525
Right superior temporal	−0.013	−1.35	0.176	0.383
Right supramarginal	−0.001	−0.11	0.914	0.958
Right frontal pole	−0.020	−1.96	0.050	0.271
Right temporal pole	−0.014	−1.52	0.129	0.377
Right transverse temporal	−0.005	−0.52	0.606	0.732
Right insula	−0.009	−0.89	0.375	0.525
Left cerebellum cortex	−0.022	−1.83	0.068	0.282
Left thalamus proper	−0.016	−1.49	0.137	0.377
Left caudate	−0.020	−1.93	0.053	0.271
Left putamen	−0.010	−0.97	0.333	0.525
Left pallidum	−0.010	−0.91	0.363	0.525
Brain stem	−0.030	−2.57	0.010	0.203
Left hippocampus	−0.027	−2.32	0.020	0.203
Left amygdala	0.002	0.14	0.890	0.944
Left accumbens area	−0.015	−1.49	0.136	0.377
Left ventral diencephalon	−0.030	−2.81	0.005	0.203
Right cerebellum cortex	−0.022	−1.95	0.052	0.271
Right thalamus proper	−0.017	−1.55	0.122	0.377
Right caudate	−0.022	−2.12	0.034	0.228
Right putamen	−0.009	−0.87	0.384	0.525
Right pallidum	−0.003	−0.29	0.773	0.851
Right hippocampus	−0.025	−2.23	0.026	0.203
Right amygdala	−0.010	−0.93	0.355	0.525
Right accumbens area	−0.005	−0.46	0.647	0.741
Right ventral diencephalon	−0.022	−2.06	0.040	0.249

### 3.3 No Evidence of Volume Predicting Future Mania Symptoms

Finally, we examined whether regional volume at baseline predicted future mania symptoms at the second‐year follow‐up. Analyses revealed that none of the regions examined showed a significant association with future prodromal mania symptoms. While some regions did show an association at uncorrected levels (Table 4), no brain regions were significantly associated with prodromal mania symptoms at the 2‐year follow‐up following FDR correction for multiple comparisons.

**TABLE 4:** Associations between second‐year prodromal mania symptoms and baseline gray matter volume in 68 cortical regions and 19 subcortical regions.

	*β*	*z*	*p*	*p*_fdr_
Left banks of superior temporal sulcus	0.005	0.35	0.726	0.876
Left caudal anterior cingulate	−0.009	−0.71	0.477	0.775
Left caudal middle frontal	−0.006	−0.47	0.637	0.840
Left cuneus	−0.018	−1.58	0.113	0.339
Left entorhinal	−0.016	−1.30	0.195	0.471
Left fusiform	−0.038	−2.57	0.010	0.137
Left inferior parietal	0.009	0.70	0.484	0.775
Left inferior temporal	−0.015	−1.04	0.299	0.591
Left isthmus cingulate	−0.003	−0.23	0.820	0.939
Left lateral occipital	−0.033	−2.33	0.020	0.152
Left lateral orbitofrontal	−0.012	−0.73	0.465	0.775
Left lingual	−0.007	−0.50	0.620	0.830
Left medial orbitofrontal	0.003	0.20	0.843	0.945
Left middle temporal	−0.001	−0.08	0.939	0.950
Left parahippocampal	−0.020	−1.69	0.092	0.320
Left paracentral	−0.015	−1.15	0.252	0.522
Left pars opercularis	0.002	0.15	0.880	0.945
Left pars orbitalis	−0.007	−0.52	0.605	0.822
Left pars triangularis	0.009	0.72	0.475	0.775
Left pericalcarine	−0.002	−0.16	0.874	0.945
Left postcentral	−0.033	−2.31	0.021	0.152
Left posterior cingulate	−0.022	−1.65	0.099	0.329
Left precentral	−0.039	−2.67	0.008	0.137
Left precuneus	−0.018	−1.22	0.224	0.494
Left rostral anterior cingulate	−0.014	−1.04	0.298	0.591
Left rostral middle frontal	−0.022	−1.48	0.139	0.383
Left superior frontal	−0.021	−1.37	0.172	0.428
Left superior parietal	−0.023	−1.77	0.077	0.320
Left superior temporal	−0.026	−1.83	0.067	0.307
Left supramarginal	−0.030	−2.16	0.031	0.180
Left frontal pole	−0.004	−0.34	0.735	0.876
Left temporal pole	−0.025	−2.12	0.034	0.185
Left transverse temporal	−0.030	−2.37	0.018	0.152
Left insula	−0.002	−0.12	0.907	0.948
Right banks of superior temporal sulcus	−0.001	−0.11	0.915	0.948
Right caudal anterior cingulate	−0.008	−0.64	0.525	0.782
Right caudal middle frontal	−0.004	−0.34	0.733	0.876
Right cuneus	−0.021	−1.72	0.085	0.320
Right entorhinal	−0.033	−2.65	0.008	0.137
Right fusiform	−0.023	−1.62	0.106	0.329
Right inferior parietal	−0.002	−0.15	0.878	0.945
Right inferior temporal	−0.041	−2.75	0.006	0.137
Right isthmus cingulate	0.005	0.38	0.704	0.876
Right lateral occipital	−0.036	−2.55	0.011	0.137
Right lateral orbitofrontal	−0.004	−0.24	0.807	0.936
Right lingual	−0.005	−0.41	0.680	0.870
Right medial orbitofrontal	0.021	1.39	0.163	0.417
Right middle temporal	−0.038	−2.39	0.017	0.152
Right parahippocampal	0.000	−0.01	0.992	0.992
Right paracentral	−0.002	−0.12	0.904	0.948
Right pars opercularis	−0.012	−0.94	0.350	0.648
Right pars orbitalis	−0.019	−1.45	0.148	0.390
Right pars triangularis	0.007	0.57	0.569	0.813
Right pericalcarine	−0.009	−0.69	0.490	0.775
Right postcentral	−0.025	−1.70	0.090	0.320
Right posterior cingulate	−0.010	−0.68	0.499	0.775
Right precentral	−0.038	−2.70	0.007	0.137
Right precuneus	−0.022	−1.47	0.141	0.383
Right rostral anterior cingulate	−0.011	−0.84	0.401	0.727
Right rostral middle frontal	−0.002	−0.17	0.866	0.945
Right superior frontal	−0.019	−1.24	0.214	0.494
Right superior parietal	−0.022	−1.63	0.103	0.329
Right superior temporal	−0.005	−0.36	0.719	0.876
Right supramarginal	−0.007	−0.54	0.589	0.813
Right frontal pole	−0.023	−1.85	0.065	0.307
Right temporal pole	−0.007	−0.63	0.530	0.782
Right transverse temporal	−0.022	−1.74	0.081	0.320
Right insula	−0.030	−2.00	0.045	0.230
Left cerebellum cortex	−0.036	−2.31	0.021	0.152
Left thalamus proper	−0.050	−3.08	0.002	0.137
Left caudate	−0.024	−1.73	0.083	0.320
Left putamen	−0.017	−1.19	0.233	0.494
Left pallidum	−0.010	−0.71	0.479	0.775
Brain stem	−0.017	−1.01	0.313	0.592
Left hippocampus	−0.010	−0.65	0.516	0.782
Left amygdala	−0.014	−1.02	0.307	0.592
Left accumbens area	−0.001	−0.09	0.928	0.950
Left ventral diencephalon	−0.021	−1.21	0.228	0.494
Right cerebellum cortex	−0.034	−2.16	0.030	0.180
Right thalamus proper	−0.036	−2.23	0.026	0.174
Right caudate	−0.008	−0.54	0.589	0.813
Right putamen	−0.023	−1.56	0.120	0.348
Right pallidum	−0.008	−0.57	0.572	0.813
Right hippocampus	−0.018	−1.21	0.226	0.494
Right amygdala	0.007	0.45	0.652	0.847
Right accumbens area	−0.010	−0.69	0.488	0.775
Right ventral diencephalon	−0.005	−0.30	0.763	0.897

## 4 Discussion

We examined associations between prodromal mania symptoms and brain volume in a large sample of children and found that the neurostructural differences associated with these symptoms are apparent in late childhood. After adjusting for age, sex, SES, scanner model, and medication use and applying correction for multiple comparisons, higher mania symptoms were associated with smaller volume across all regions at baseline. However, these relationships no longer held once total intracranial volume was taken into account, suggesting these associations may reflect global differences in overall brain size rather than region‐specific alterations. Furthermore, baseline volume did not predict mania symptoms at the second‐year follow‐up. Together, these results suggest that while early prodromal mania symptoms are related to structural brain differences, the effects appear nonspecific to individual regions and may be largely attributable to overall brain volume.

Our findings are broadly consistent with prior research documenting smaller gray matter volumes in youth with bipolar disorder and in those at elevated risk. Similar to studies reporting reduced cortical volume in frontal regions (Lim et al. 2013; Roberts et al. 2022) and smaller amygdala volumes in youth with bipolar disorder (Förster et al. 2023; Long et al. 2023), we also observed that higher prodromal mania symptoms were associated with smaller regional volumes across multiple areas. Although most prior studies have relied on clinical or high‐risk samples, our results extend this literature by showing comparable directions of associations in a large, community‐based cohort of children who had not yet received a diagnosis. This suggests that structural correlates of mania symptoms may be detectable even before the onset of the first manic or hypomanic episode, supporting the idea that such neurobiological differences emerge early in development and may represent dimensional vulnerability markers along the bipolar spectrum.

Notably, the associations between mania symptoms and smaller regional volumes did not persist after adjusting for total intracranial volume, indicating that the effects we observed were global rather than localized to specific cortical or subcortical regions. This is consistent with prior work showing that children with bipolar disorder may have smaller overall cerebral volume (Frazier, Ahn, et al. 2005), suggesting that brain‐wide structural differences may be important predictors of symptom expression during development. One interpretation is that mania‐related neurostructural alterations in childhood reflect a general delay or difference in neurodevelopmental processes, such as synaptic pruning, cortical maturation, or global brain growth, rather than disruptions of particular circuits. However, it is also important to note that these associations may not be unique to mania, as smaller global brain volumes have also been linked to general psychopathology across multiple disorders (Durham et al. 2023, 2021; Kaczkurkin et al. 2020, 2019). Thus, this global pattern could suggest that prodromal mania symptoms in childhood are tapping into broad neurostructural differences evident across mental health conditions, which highlights the importance of considering global brain growth trajectories in models of early psychopathology.

Contrary to our hypotheses, baseline brain volume did not predict mania symptoms at the 2‐year follow‐up. This null result suggests that structural differences at ages 9 and 10 years may not be robust predictors of symptom trajectories over short developmental windows. One possibility is that structural alterations observed at this early age reflect transient maturational variability rather than stable risk markers. Another possibility is that brain–behavior relationships become more specific and predictive closer to the age of peak risk for manic episodes, which typically occurs in late adolescence or early adulthood (American Psychiatric Association 2022; Goldstein et al. 2017). Alternatively, symptom progression may be more strongly shaped by dynamic factors such as functional brain changes, environmental stressors, or gene–environment interactions, which could interact with structural differences later in development. The ongoing longitudinal follow‐up of the ABCD Study cohort into adolescence and early adulthood will be critical for determining whether neurostructural markers acquire greater predictive utility as prodromal symptoms consolidate and clinical episodes emerge.

This study offers several notable strengths. The ABCD Study's large, community‐based sample provides substantial statistical power to detect subtle effects and captures a wide spectrum of mania symptoms beyond clinical thresholds. By avoiding a narrow focus on children with the most severe symptoms, we reduced the risk of inflated effect sizes that can occur in case–control designs. The restricted age range of the sample also minimized the confounding effects of normative brain development. Together, these features enhance the generalizability of our findings to the broader population. At the same time, certain limitations should be acknowledged. Mania symptoms were assessed exclusively via caregiver report, which may introduce bias or reduce sensitivity to subtle symptom expression. In addition, while our effects were small, this pattern is common in large‐scale neuroimaging studies of brain–behavior relationships (Paulus and Thompson 2019). Small but reliable effects may nonetheless be informative at the population level, though their clinical utility remains to be determined.

This study provides a valuable step forward in understanding the link between mania symptoms and structural differences during late childhood. However, further work should be done to track the relationship between mania symptoms and brain structure from late childhood onward to better understand when this association stabilizes and/or diverges. Future work could also supplement the current findings by investigating other early indicators of bipolar disorder, such as inflammation. Prior work has implicated several hematological indices (including neutrophil count, platelet count, mean platelet volume, neutrophil‐to‐lymphocyte ratio, platelet‐to‐lymphocyte ratio, and monocyte‐to‐lymphocyte ratio) as potential predictors of mood state, with platelet‐to‐lymphocyte ratio in particular suggested as an independent predictor of mania and hypomania (Fusar‐Poli et al. 2021). Given the growing evidence that neuroinflammatory processes may influence brain structure and function, incorporating such markers into longitudinal neuroimaging studies could clarify potential mechanistic links between inflammation and brain changes associated with mania risk.

An additional important avenue for future research involves the integration of detailed treatment‐related variables into longitudinal studies of brain development in youth with prodromal mania symptoms. Although our current analyses controlled for baseline medication use, we did not have the ability to assess the nuanced clinical factors of treatment response or adherence over time. Prior work in mood disorders has shown that illness severity and treatment side effects are significant predictors of nonadherence, which in turn can influence clinical outcomes (Pompili et al. 2009). These issues are also likely to be highly relevant for youth at risk for bipolar disorder, as early treatment initiation and adherence may interact with neurodevelopmental processes. For example, inadequate treatment adherence during critical developmental windows could exacerbate mood symptoms and potentially contribute to neuroanatomical differences through mechanisms such as heightened allostatic load, recurrent mood episodes, or prolonged exposure to mood instability. Conversely, effective and sustained treatment engagement may help normalize developmental brain trajectories or mitigate progressive structural changes. Future studies that combine longitudinal neuroimaging with detailed treatment histories, adherence measures, and side effect profiles could clarify the extent to which these clinical variables moderate the relationship between prodromal mania symptoms and brain structure.

In summary, this study examined the neuroanatomical correlates of prodromal mania symptoms in a large sample of children, revealing that higher baseline mania symptoms were associated with smaller global gray matter volumes across cortical and subcortical regions. These associations did not persist when total intracranial volume was included as a covariate, further underscoring the importance of considering global brain metrics in studies of mood symptomatology. Taken together, our findings point to smaller brain size as a potential neurodevelopmental marker of early mania risk, emphasizing the need for longitudinal investigations that can disentangle causal pathways, identify early intervention targets, and ultimately improve outcomes for youth at heightened risk for bipolar disorder.

## Author Contributions

**Camille Archer**: conceptualization, formal analysis, visualization, writing – original draft, writing – review and editing. **Amy Milewski**: conceptualization, formal analysis, visualization, writing – original draft, writing – review and editing. **Hee Jung Jeong**: writing – review and editing. **Gabrielle E. Reimann**: writing – review and editing. **E. Leighton Durham**: writing – review and editing. **Antonia N. Kaczkurkin**: conceptualization, formal analysis, funding acquisition, methodology, resources, supervision, visualization, writing – original draft, writing – review and editing.

## Conflicts of Interest

The authors declare no conflicts of interest.

## Ethics Statement

Vanderbilt University's Institutional Review Board approved the use of this publicly available, de‐identified dataset.

## Consent

Caregivers in the ABCD Study provided informed consent and minors provided informed assent.

## Peer Review

The peer review history for this article is available at https://publons.com/publon/10.1002/brb3.70894.

## Data Availability

The National Institute of Mental Health Data Archive (https://nda.nih.gov/abcd) provides access to the ABCD Study data. To access the current analyses’ scripts with a detailed description, visit https://github.com/VU‐BRAINS‐lab/Archer_Mania_Volume.

## References

[brb370894-bib-0001] Abé, C. , C. J. Ekman , C. Sellgren , P. Petrovic , M. Ingvar , and M. Landén . 2016. “Cortical Thickness, Volume and Surface Area in Patients With Bipolar Disorder Types I and II.” Journal of Psychiatry and Neuroscience 41, no. 4: 240–250. 10.1503/jpn.150093. 26645741 PMC4915933

[brb370894-bib-0002] American Psychiatric Association . 2022. Diagnostic and Statistical Manual of Mental Disorders. 5th ed., text revision. American Psychiatric Association. 10.1176/appi.books.9780890425787.

[brb370894-bib-0003] Angelescu, I. , S. P. Brugger , F. Borgan , S. J. Kaar , and O. D. Howes . 2021. “The Magnitude and Variability of Brain Structural Alterations in Bipolar Disorder: A Double Meta‐Analysis of 5534 Patients and 6651 Healthy Controls.” Journal of Affective Disorders 291: 171–176. 10.1016/j.jad.2021.04.090. 34038834

[brb370894-bib-0004] Axelson, D. A. , B. Birmaher , M. A. Strober , et al. 2011. “Course of Subthreshold Bipolar Disorder in Youth: Diagnostic Progression from Bipolar Disorder Not Otherwise Specified.” Journal of the American Academy of Child and Adolescent Psychiatry 50, no. 10: 1001.e3–1016.e3. www.jaacap.org. 21961775 10.1016/j.jaac.2011.07.005 PMC3185249

[brb370894-bib-0005] Barch, D. M. , M. D. Albaugh , S. Avenevoli , et al. 2018. “Demographic, Physical and Mental Health Assessments in the Adolescent Brain and Cognitive Development Study: Rationale and Description.” Developmental Cognitive Neuroscience 32: 55–66. 10.1016/j.dcn.2017.10.010. 29113758 PMC5934320

[brb370894-bib-0006] Casey, B. J. , T. Cannonier , M. I. Conley , et al. 2018. “The Adolescent Brain Cognitive Development (ABCD) Study: Imaging Acquisition Across 21 Sites.” Developmental Cognitive Neuroscience 32: 43–54. 10.1016/j.dcn.2018.03.001. 29567376 PMC5999559

[brb370894-bib-0007] Conroy, S. K. , M. M. Francis , and L. A. Hulvershorn . 2018. “Identifying and Treating the Prodromal Phases of Bipolar Disorder and Schizophrenia.” Current Treatment Options in Psychiatry 5, no. 1: 113–128. 10.1007/s40501-018-0138-0. 30364516 PMC6196741

[brb370894-bib-0008] Correll, C. U. , M. Hauser , J. B. Penzner , et al. 2014. “Type and Duration of Subsyndromal Symptoms in Youth With Bipolar I Disorder Prior to Their First Manic Episode.” Bipolar Disorders 16, no. 5: 478–492. 10.1111/bdi.12194. 24597782 PMC4186919

[brb370894-bib-0009] De Pablo, G. S. , D. Guinart , B. A. Cornblatt , et al. 2020. “Demographic and Clinical Characteristics, Including Subsyndromal Symptoms Across Bipolar‐Spectrum Disorders in Adolescents.” Journal of Child and Adolescent Psychopharmacology 30, no. 4: 222–234. 10.1089/cap.2019.0138. 32083495 PMC7232658

[brb370894-bib-0010] Desikan, R. S. , F. Ségonne , B. Fischl , et al. 2006. “An Automated Labeling System for Subdividing the Human Cerebral Cortex on MRI Scans Into Gyral Based Regions of Interest.” NeuroImage 31, no. 3: 968–980. 10.1016/j.neuroimage.2006.01.021. 16530430

[brb370894-bib-0011] Dickstein, D. P. , M. P. Milham , A. C. Nugent , et al. 2005. “Frontotemporal Alterations in Pediatric Bipolar Disorder: Results of a Voxel‐Based Morphometry Study.” Archives of General Psychiatry 62, no. 7: 734–741. 10.1001/ARCHPSYC.62.7.734. 15997014

[brb370894-bib-0012] Durham, E. L. , K. Ghanem , A. J. Stier , et al. 2023. “Multivariate Analytical Approaches for Investigating Brain‐Behavior Relationships.” Frontiers in Neuroscience 17: 1175690. 10.3389/fnins.2023.1175690. 37583413 PMC10423877

[brb370894-bib-0013] Durham, E. L. , H. J. Jeong , T. M. Moore , et al. 2021. “Association of Gray Matter Volumes With General and Specific Dimensions of Psychopathology in Children.” Neuropsychopharmacology 46, no. 7: 1333–1339. 10.1038/s41386-020-00952-w. 33479512 PMC8134562

[brb370894-bib-0014] Fischl, B. , D. Salat , E. Busa , M. Albert , M. Dieterich , and C. Haselgrove . 2002. “Whole Brain Segmentation: Automated Labeling of Neuroanatomical Structures in the Human Brain.” Neuron 33, no. 3: 341–355. 10.1016/S0896-6273(02)00569-X. 11832223

[brb370894-bib-0015] Förster, K. , R. H. Horstmann , U. Dannlowski , J. Houenou , and P. Kanske . 2023. “Progressive Grey Matter Alterations in Bipolar Disorder Across the Life Span—A Systematic Review.” Bipolar Disorders 25, no. 6: 443–456. 10.1111/BDI.13318. 36872645

[brb370894-bib-0016] Frazier, J. A. , M. S. Ahn , S. DeJong , E. K. Bent , J. L. Breeze , and A. J. Giuliano . 2005. “Magnetic Resonance Imaging Studies in Early‐Onset Bipolar Disorder: A Critical review.” Harvard Review of Psychiatry 13, no. 3: 125–140. 10.1080/10673220591003597. 16020026

[brb370894-bib-0017] Frazier, J. A. , J. L. Breeze , N. Makris , et al. 2005. “Cortical Gray Matter Differences Identified by Structural Magnetic Resonance Imaging in Pediatric Bipolar Disorder.” Bipolar Disorders 7, no. 6: 555–569. 10.1111/j.1399-5618.2005.00258.x. 16403181 PMC2072813

[brb370894-bib-0018] Freeman, A. J. , E. A. Youngstrom , T. W. Frazier , J. K. Youngstrom , C. Demeter , and R. L. Findling . 2012. “Portability of a Screener for Pediatric Bipolar Disorder to a Diverse Setting.” Psychological Assessment 24, no. 2: 341–351. 10.1037/a0025617. 21942229 PMC3495327

[brb370894-bib-0019] Fusar‐Poli, L. , A. Natale , A. Amerio , et al. 2021. “Neutrophil‐to‐Lymphocyte, Platelet‐to‐Lymphocyte and Monocyte‐to‐Lymphocyte Ratio in Bipolar Disorder.” Brain Sciences 11, no. 1: 58. 10.3390/brainsci11010058. 33418881 PMC7825034

[brb370894-bib-0020] Gogtay, N. , A. Ordonez , D. H. Herman , et al. 2007. “Dynamic Mapping of Cortical Development Before and After the Onset of Pediatric Bipolar Illness.” Journal of Child Psychology and Psychiatry and Allied Disciplines 48, no. 9: 852–862. 10.1111/j.1469-7610.2007.01747.x. 17714370

[brb370894-bib-0021] Goldstein, B. I. , B. Birmaher , G. A. Carlson , et al. 2017. “The International Society for Bipolar Disorders Task Force Report on Pediatric Bipolar Disorder: Knowledge to Date and Directions for Future Research.” Bipolar Disorders 19, no. 7: 524–543. 10.1111/bdi.12556. 28944987 PMC5716873

[brb370894-bib-0022] Hagler, D. J. , S. N. Hatton , M. D. Cornejo , et al. 2019. “Image Processing and Analysis Methods for the Adolescent Brain Cognitive Development Study.” NeuroImage 202: 116091. 10.1016/j.neuroimage.2019.116091. 31415884 PMC6981278

[brb370894-bib-0023] Hibar, D. P. , L. T. Westlye , N. T. Doan , et al. 2018. “Cortical Abnormalities in Bipolar Disorder: An MRI Analysis of 6503 Individuals From the ENIGMA Bipolar Disorder Working Group.” Molecular Psychiatry 23, no. 4: 932–942. 10.1038/mp.2017.73. 28461699 PMC5668195

[brb370894-bib-0024] Hibar, D. P. , L. T. Westlye , T. G. M. Van Erp , et al. 2016. “Subcortical Volumetric Abnormalities in Bipolar Disorder.” Molecular Psychiatry 21, no. 12: 1710–1716. 10.1038/mp.2015.227. 26857596 PMC5116479

[brb370894-bib-0025] Kaczkurkin, A. N. , T. M. Moore , A. Sotiras , C. H. Xia , R. T. Shinohara , and T. D. Satterthwaite . 2020. “Approaches to Defining Common and Dissociable Neurobiological Deficits Associated With Psychopathology in Youth.” Biological Psychiatry 88: 51–62. 10.1016/j.biopsych.2019.12.015. 32087950 PMC7305976

[brb370894-bib-0026] Kaczkurkin, A. N. , S. S. Park , A. Sotiras , et al. 2019. “Evidence for Dissociable Linkage of Dimensions of Psychopathology to Brain Structure in Youths.” American Journal of Psychiatry 176: 1000–1009. 10.1176/appi.ajp.2019.18070835. 31230463 PMC6888993

[brb370894-bib-0027] Kaur, S. , R. B. Sassi , D. Axelson , et al. 2005. “Cingulate Cortex Anatomical Abnormalities in Children and Adolescents With Bipolar Disorder.” American Journal of Psychiatry 162, no. 9: 1637–1643. 16135622 10.1176/appi.ajp.162.9.1637

[brb370894-bib-0028] Lan, M. J. , B. T. Chhetry , M. A. Oquendo , et al. 2014. “Cortical Thickness Differences Between Bipolar Depression and Major Depressive Disorder.” Bipolar Disorders 16, no. 4: 378–388. 10.1111/bdi.12175. 24428430 PMC4047134

[brb370894-bib-0029] Lim, C. S. , R. J. Baldessarini , E. Vieta , M. Yucel , E. Bora , and K. Sim . 2013. “Longitudinal Neuroimaging and Neuropsychological Changes in Bipolar Disorder Patients: Review of the Evidence.” Neuroscience and Biobehavioral Reviews 37, no. 3: 418–435. 10.1016/j.neubiorev.2013.01.003. 23318228

[brb370894-bib-0030] Long, X. , L. Li , X. Wang , et al. 2023. “Gray Matter Alterations in Adolescent Major Depressive Disorder and Adolescent Bipolar Disorder.” Journal of Affective Disorders 325: 550–563. 10.1016/J.JAD.2023.01.049. 36669567

[brb370894-bib-0031] Maletic, V. , and C. Raison . 2014. “Integrated Neurobiology of Bipolar Disorder.” Frontiers in Psychiatry 5: 98. 10.3389/fpsyt.2014.00098. 25202283 PMC4142322

[brb370894-bib-0032] Muneer, A. 2016. “The Neurobiology of Bipolar Disorder: An Integrated Approach.” Chonnam Medical Journal 52, no. 1: 18–37. 10.4068/cmj.2016.52.1.18. 26865997 PMC4742607

[brb370894-bib-0033] Paulus, M. P. , and W. K. Thompson . 2019. “The Challenges and Opportunities of Small Effects: The New Normal in Academic Psychiatry.” JAMA Psychiatry 76, no. 4: 353–354. 10.1001/jamapsychiatry.2018.4540. 30810720

[brb370894-bib-0034] Pompili, M. , G. Serafini , A. Del Casale , et al. 2009. “Improving Adherence in Mood Disorders: The Struggle Against Relapse, Recurrence and Suicide Risk.” Expert Review of Neurotherapeutics 9, no. 7: 985–1004. 10.1586/ERN.09.62. 19589049

[brb370894-bib-0035] Roberts, G. , R. Lenroot , B. Overs , et al. 2022. “Accelerated Cortical Thinning and Volume Reduction Over Time in Young People at High Genetic Risk for Bipolar Disorder.” Psychological Medicine 52, no. 7: 1344–1355. 10.1017/S0033291720003153. 32892764

[brb370894-bib-0036] Skjelstad, D. V. , U. F. Malt , and A. Holte . 2010. “Symptoms and Signs of the Initial Prodrome of Bipolar Disorder: A Systematic Review.” Journal of Affective Disorders 126, no. 1–2: 1–13. 10.1016/j.jad.2009.10.003. 19883943

[brb370894-bib-0037] Toma, S. , A. H. Islam , A. W. S. Metcalfe , et al. 2019. “Cortical Volume and Thickness Across Bipolar Disorder Subtypes in Adolescents: A Preliminary Study.” Journal of Child and Adolescent Psychopharmacology 29, no. 2: 141–151. 10.1089/cap.2017.0137. 30359542

[brb370894-bib-0038] Van Meter, A. , C. U. Correll , W. Ahmad , M. Dulin , and E. Saito . 2021. “Symptoms and Characteristics of Youth Hospitalized for Depression: Subthreshold Manic Symptoms Can Help Differentiate Bipolar From Unipolar Depression.” Journal of Child and Adolescent Psychopharmacology 31, no. 8: 545–552. 10.1089/cap.2021.0057. 34637626 PMC10331145

[brb370894-bib-0039] Volkow, N. D. , G. F. Koob , R. T. Croyle , et al. 2018. “The Conception of the ABCD Study: From Substance Use to a Broad NIH Collaboration.” Developmental Cognitive Neuroscience 32: 4–7. 10.1016/j.dcn.2017.10.002. 29051027 PMC5893417

[brb370894-bib-0040] Wang, X. , Q. Luo , F. Tian , et al. 2019. “Brain Grey‐Matter Volume Alteration in Adult Patients With Bipolar Disorder Under Different Conditions: A Voxel‐Based Meta‐Analysis.” Journal of Psychiatry and Neuroscience 44, no. 2: 89–101. 10.1503/jpn.180002. 30354038 PMC6397036

[brb370894-bib-0041] Wise, T. , J. Radua , E. Via , et al. 2017. “Common and Distinct Patterns of Grey‐Matter Volume Alteration in Major Depression and Bipolar Disorder: Evidence From Voxel‐Based Meta‐Analysis.” Molecular Psychiatry 22, no. 10: 1455–1463. 10.1038/mp.2016.72. 27217146 PMC5622121

[brb370894-bib-0042] Youngstrom, E. A. , T. W. Frazier , C. Demeter , J. R. Calabrese , and R. L. Findling . 2008. “Developing a 10‐Item Mania Scale From the Parent General Behavior Inventory for Children and Adolescents.” Journal of Clinical Psychiatry 69, no. 5: 831–839. 10.4088/JCP.V69N0517. 18452343 PMC2777983

